# Effectiveness of Interventions, Programs and Strategies for Gender-based Violence Prevention in Refugee Populations: An Integrative Review

**DOI:** 10.1371/currents.dis.3a465b66f9327676d61eb8120eaa5499

**Published:** 2016-04-19

**Authors:** Hannah Tappis, Jeffrey Freeman, Nancy Glass, Shannon Doocy

**Affiliations:** Johns Hopkins Bloomberg School of Public Health, Baltimore, MD, USA

## Abstract

Background: Gender based violence (GBV) remains one of the most serious threats to the health and safety of women and girls worldwide. The problem is even more pronounced in refugee populations where women and girls are at increased risk of violence. In 2015, UNHCR reported the highest number of forcibly displaced people in recorded history. Despite growing need, there have been few rigorous evaluations of interventions aimed at primary GBV prevention and no systematic reviews of GBV prevention efforts specifically focused on refugee populations; reviews to date have primarily examined prevention of conflict related sexual violence, with very limited focus on other forms of GBV such as intimate partner violence

Methods: This study reviewed the scientific literature addressing strategies for primary prevention of GBV and their effectiveness among refugee populations over the past ten years (2006 to 2015). Narrative content analysis methods were used to extract findings related to prevention activities/programs recommended by the global humanitarian community, such as sociocultural norms change, rebuilding family and community support structures, improving accountability systems, designing effective services and facilities, working with formal and traditional legal systems, monitoring and documenting GBV, and/or engaging men and boys in GBV prevention and response.

Results: Study findings indicate that a range of GBV prevention activities recommended by the global humanitarian community are currently being applied in a variety of settings. However, there remains a limited body of evidence on the effectiveness of GBV prevention programs, interventions, and strategies, especially among refugee populations.

Conclusion: Commonly agreed upon standards or guidelines for evaluation of GBV prevention programming, and publication of evaluations conducted using these guidelines, could assist humanitarian stakeholders to build and disseminate an evidence base of effective GBV prevention interventions, programs and strategies. Evaluation of GBV prevention efforts, especially among refugee populations, must be given higher priority to justify continuation or revision of recommended GBV activities/programs being implemented in diverse humanitarian settings.

## Background

Gender-based violence (GBV) remains one of the most prevalent and persistent issues facing women and girls globally 1. The United Nations High Commissioner for Human Rights’ Committee on the Elimination of Discrimination against Women (CEDAW) defines GBV as “violence that is directed against a woman because she is a woman or that affects women disproportionately” 2. The UN General Assembly Declaration on the Elimination of Violence Against Women (1993) expanded the scope of GBV to encompass physical, sexual and psychological violence, including threats and coercion occurring within families, in the general community, or condoned by the State 3.

The World Health Organization (WHO) estimates that 35% of women experience some kind of physical and/or sexual violence at some point in their lives 4. The problem is even more pronounced in refugee populations where women and girls are at increased risk of GBV 5 ^,^ 6 ^,^ 7. Several factors have been cited as causing increased risk of GBV including extreme poverty, minority status, lack of access to food and water, and disrupted family and community support systems, among others 8.

The displaced population at risk of GBV continues to grow in parallel with the size of the population displaced by conflict which is presently estimated at 59.5 million forcibly displaced worldwide, which is the highest levels recorded since the end of World War II 9. Record numbers of displacement were reported in 2013 and 2014 and it appears that 2015 reports will continue with the pattern of increases in the size of population displaced by conflict 10. Much of the displacement has been driven by the continued violent conflicts in Afghanistan, Democratic Republic of Congo, Iraq, and Syria among others 11. A recent commentary in The Lancet discusses the issue of violence against Syrian female refugees, and cites a catastrophic trend of using sexual violence as a tactic of war 12. This is not a new issue, as GBV and particularly sexual violence have long been understood to be a direct consequence of armed conflict 13 ^,^ 14. The impact of GBV among refugee populations varies by region and context, but may include increased risk of HIV and other sexually transmitted infections (STIs) as indicated by studies in DR Congo; depression and post-traumatic stress disorder documented throughout Sub-Saharan Africa; as well as short-and long term health, economic and social sequelae for individuals, families and communities documented in a variety of geographic locations globablly 15 ^,^ 16 ^,^ 17 ^,^ 18 ^,^ 19.

Six types of GBV prevention programming identified in UNHCR’s 2003 Sexual and Gender-Based Violence against Refugees, Returnees and Internally Displaced Persons: Guidelines for Prevention and Response are as follows 20:

(1) Transforming socio-cultural norms, with an emphasis on empowering women and girls

(2) Rebuilding family and community structures and support systems

(3) Creating conditions to improve accountability systems

(4) Designing effective services and facilities

(5) Working with formal and traditional legal systems

(6) Assessment, monitoring, and documentation of GBV

The guidelines recommend involving men as a key strategy for transforming socio-cultural norms and highlight the importance of equal participation by women, men, girls, and boys in planning, implementing, monitoring, and evaluating programs. Over the last decade, there has been increasing attention to the fact that men’s roles and relationships to GBV in conflict and post-conflict situations include that of perpetrators, victims, witnesses and agents of change, and that male engagement is an essential component of GBV prevention and response programming in humanitarian settings 21. These seven types of programming reflect recommendations made by the global humanitarian community, and are all examples of primary prevention efforts, that is efforts to reduce the number of new incidents of violence by intervening before any violence occurs. This approach contrasts with other prevention efforts that seek to reduce the harmful consequences of an act of violence after it has occurred, or to prevent further acts of violence from occurring once violence has been identified 22.

Although there has been an increase in the number of programs in refugee populations to prevent and respond to GBV, particularly sexual violence against women and girls, there remains a general lack of evidence regarding the effectiveness of these efforts in preventing diverse forms of GBV, and a lack of evaluation of efforts outside of conflict related sexual violence 23 ^,^ 24. There have been several review papers published examining the efficacy of GBV prevention efforts, but none have focused specifically on refugee settings where the risks to women and girls may be higher. For example, a systematic review of reviews published in 2014 by Arango et al., examined GBV prevention measures globally, but focused primarily on efforts in high-income countries, where the majority of evaluations have been concentrated, and did not include prevention efforts focused on refugee populations 25. Another review by Spangaro et al. published in 2015, examined interventions in settings with armed conflict, including some with refugee populations; however, the authors examined sexual violence exclusively, which is just one type of GBV that women and girls may experience^26^.

Recent evaluations of humanitarian programs have also highlighted the need for evidence on effective GBV prevention strategies. For example, a recent evaluation in Syria of the implementation of the Inter-Agency Standing Committee (IASC) Guidelines for Gender-Based Violence Interventions in Humanitarian Settings found that IASC guidelines were not well known nor being used in programmatic practice, and while there have been ‘high level’ statements that prevention and response efforts require involvement at many levels and types of actors, there is lack of practical guidance about how to incorporate GBV prevention programming across diverse humanitarian sectors, such as health, water and sanitation, education, and livelihood programs 27. In response to the continued need for evidence on effective GBV prevention strategies for refugee populations, and in an effort to expand review beyond just conflict related sexual violence, this study aims to provide a review of publications related to GBV prevention strategies recommended by the global humanitarian community for refugee populations over the last ten years.

## Methods

An integrative review of scientific literature on GBV prevention was initially conducted in 2013 and later updated in October 2015 using the following databases: Pubmed, Scopus and Web of Science. The search was limited to studies published in English over the previous ten years (i.e. 2006 to 2015). Key search terms were identified through a preliminary review of relevant literature in consultation with a public health information specialist at the Johns Hopkins University Welch Medical Library. A summary of search terms, databases, inclusion criteria and other information on the selection strategy is presented in Table 1.

**Figure:**
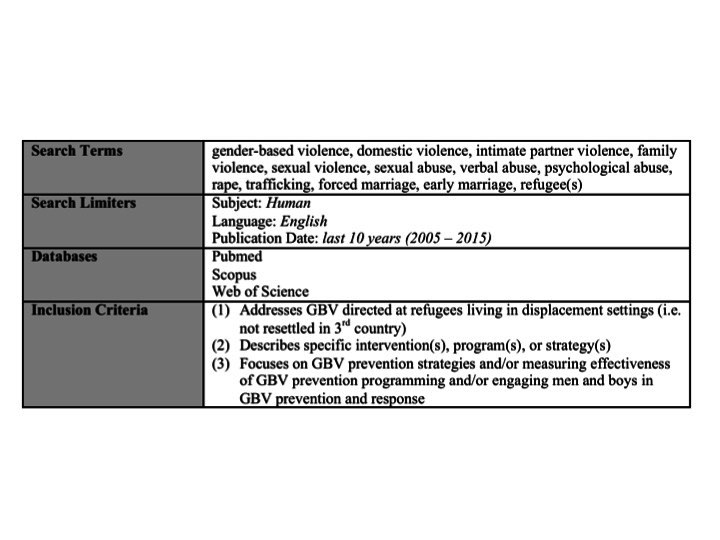
Table 1. Summary of systematic review search terms, search limiters, databases, and inclusion criteria.

**Screening of Literature**

Following removal of duplicate articles (i.e. articles listed in multiple databases), two independent reviewers conducted a multi-stage title and abstract screening process to determine each study's eligibility for inclusion in the review. In the first round of screening, any study that met the three inclusions criteria were included for further review, regardless of study design. To ensure no potentially relevant studies were omitted, titles and abstracts of excluded studies were then re-screened and any studies focused on specific GBV prevention interventions, programs or strategies among non-refugee populations in crisis-prone, crisis-affected, or low-resource settings that could inform GBV prevention in refugee populations were included (including studies examining GBV prevention efforts for internally displaced populations). All studies that met content-based inclusion criteria were included, regardless of the study design, quality or potential risk of bias.

**Data Extraction and Synthesis**

All included studies were reviewed in detail and coded according to their relevance to the seven types of primary prevention programming mentioned above: transforming socio-cultural norms, with an emphasis on empowering women and girls; rebuilding family and community structures and support systems; creating conditions to improve accountability systems; designing effective services and facilities; working with formal and traditional legal systems; assessment, monitoring, and documentation of GBV; and engaging men and boys in GBV prevention and response.

Key findings related to each type of primary prevention programming were analysed separately using narrative content analysis techniques, and then results combined to provide an overall synthesis of publications related to GBV prevention in refugee populations.

**Ethical Review**

This review did not involve any primary data collection and thus did not require review by an Institutional Review Board or ethics committee.

## Results

A total of 618 articles were identified. After removal of duplicates, 393 articles were included in the title/abstract review. Of the 393 articles initially identified for review, only ten met all three inclusion criteria, including: 4 reviews, five qualitative studies, and one mixed methods stud. In addition, thirteen articles on GBV prevention in non-refugee populations were identified as relevant (see previously defined screening methods) and were included in full text review (Figure 1), including: 1 review, five quantitative studies, four qualitative studies, 2 mixed-methods studies, and 1 commentary.

**Figure:**
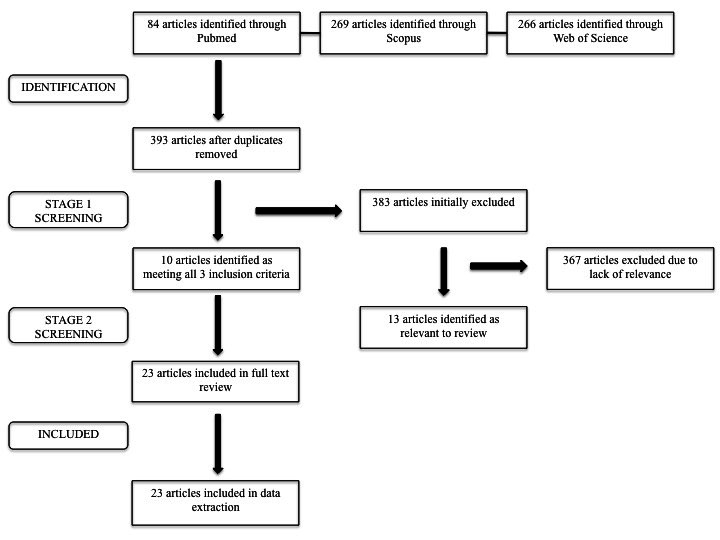
Literature review search indicating process of selection for GBV prevention articles included in review.

A total of twenty-three peer-reviewed articles were identified for data extraction and included in the review (Table 2,). Evaluation of peer-reviewed articles found that the body of evidence on GBV prevention was limited, particularly in refugee populations. Most studies addressed gaps in prevention and response programming or designing of effective services (7 of 10 studies). Three of ten studies in displaced populations addressed improving accountability while only one study addressed monitoring and documentation of GBV, and one study discussed working with legal systems. Articles reviewed in non-displaced settings included process and impact evaluations of interventions designed to transform social norms (11 of 13 studies), designing effective services (2 of 13 studies), monitoring and documentation (1 of 13), working with legal systems (1 of 13 studies), and male engagement (6 of 13 studies).

A total of twenty-three peer-reviewed articles were identified for data extraction and included in the review (Table 2,). Evaluation of peer-reviewed articles found that the body of evidence on GBV prevention was limited, particularly in refugee populations. Most studies addressed gaps in prevention and response programming or designing of effective services (7 of 10 studies). Three of ten studies in displaced populations addressed improving accountability while only one study addressed monitoring and documentation of GBV, and one study discussed working with legal systems. Articles reviewed in non-displaced settings included process and impact evaluations of interventions designed to transform social norms (11 of 13 studies), designing effective services (2 of 13 studies), monitoring and documentation (1 of 13), working with legal systems (1 of 13 studies), and male engagement (6 of 13 studies).

**Table:** Table 2. Summary details of articles on GBV prevention identified for data extraction and included in review.

Study / Type	Population / Settings / Unit of Analysis	Article Summary
Marsh 2006^28^ / Review	Displaced populations / Humanitarian emergency settings / Female Adult & Female Child	Reviews available published and unpublished data to illustrate the extent of sexual violence in humanitarian emergencies and the lack of a sufficiently robust response to the problem.
Pronyk 2006^31^ / Original research, quantitative	Non-displaced populations / Rural women in Limpopo Province, South Africa / Female Adult	Describes a cluster randomized trial to evaluate the Intervention with Microfinance for AIDS & Gender Equity (IMAGE), a package that combined microfinance with a participatory gender and HIV training curriculum to rural women in Limpopo Province, South Africa. Effect estimates suggest that, relative to a matched comparison group, IMAGE participants experienced a 55% (9%; 77%) reduction in the past year experience of physical and/or sexual violence by an intimate partner.
Chynoweth 2008^29^ / Original research, qualitative	Displaced populations / Iraqi refugees in Jordan / Female Adult	Explores the priority reproductive health needs and service gaps faced by Iraqi refugees in Jordan following a 2007 Women’s Commission field mission to Amman, Jordan, which found that Iraqi refugees have limited access to reproductive health services. Findings included gaps in adherence to international standards for reproductive health in emergencies, including prevention of and response to sexual violence.
Henttonen 2008^30^ / Original research, mixed	Displaced populations / GBV survivors in Northern Uganda / Female Adult	Presents a study designed to examine the status of health services available for GBV survivors in Northern Uganda, assess available GBV programs and identify gaps and challenges in the provision of services for survivors, using the IASC guidelines on GBV in humanitarian settings as an analytical framework. Conclusions call for more evidence on the effectiveness and sustainability of GBV programs.
Hargreaves 2009^34^ / Original research, mixed	Non-displaced populations / Rural settings in South Africa / Female Adult	Describes the process evaluation of an intervention combining microfinance with gender and HIV training for the prevention of intimate partner violence in South Africa. It found that microfinance and gender/HIV training were feasible to deliver and acceptable to most clients. Although participation in community mobilization was high for some clients, others experienced barriers to collective action, which may help explain lack of intervention effects among household/community members.
Barker 2010^32^ / Review	Non-displaced populations / Studies of men and boys in a variety of geographical areas / Male Adult & Male Child	Describes a review of 58 evaluation studies of programs with men and boys in sexual and reproductive health; father involvement; gender-based violence; maternal, newborn and child health; and gender socialization more broadly. The evidence from these studies indicates that programs incorporating a gender-transformative approach and promoting gender-equitable relationships between men and women are more effective in producing behavior change than narrowly focused interventions, as are programs which reach beyond the individual to the social context.
Horn 2010^33^ / Original research, qualitative	Displaced populations / Kakuma refugee camp in Kenya / Female Adult	Presents a qualitative study designed to explore how refugees living in Kakuma camp in Kenya talk about the way the intersection of community responses to intimate partner violence and the formal response systems established by UNHCR and its implementing partners. Findings from focus group discussion involving a total of 157 refugees suggest that while community responses to intimate partner violence do not necessarily result in the protection of women, women are reluctant to report their cases to UNHCR and its implanting agencies.
Jan 2011^35^ / Original research, quantitative	Non-displaced populations / Rural settings in South Africa / Female Adult	Describes the evaluation of the cost-effectiveness of an intervention combining microfinance with gender and HIV training for the prevention of intimate partner violence in South Africa. It found that the intervention was cost-effective in the trial phase and highly cost-effective in scale-up, suggesting that proven development initiatives such as microfinance represent ideal vehicles for value-adding public health interventions such as GBV prevention programming.
Ho 2011^36^ / Original research, qualitative	Displaced populations / Refugee camp in Rwanda / General	Presents findings of a study exploring refugees’ perspectives on gender-based violence and systems for holding perpetrators accountable for their actions. The study concluded that a top-down approach to GBV prevention may ironically reinforce disparities by affording the same actors that exploit community members the power to define and control monitoring, response and evaluation processes. By shifting the focus to community dialogues surrounding GBV and processes that will empower these individuals to take charge of their lives, a focus on capabilities of camp residents may swing the vertical power structure so that health and human rights are more protected.
Sikweyiya 2011^37^ / Original research, qualitative	Non-displaced populations / Interviews with 12 GBV researchers from various geographical areas / General	Presents a qualitative study gathering empirical data on the meanings of safety, the basis of ethical concerns and the nature and scope of empirical evidence on the presence of risks unique to GBV research from publications and researchers who have worked in the field. It concludes that the notion that GBV studies carry greater than minimal risk when ethics precautions are followed is based on speculation, not evidence.
Abramsky 2012^38^ / Original research, quantitative	Non-displaced populations / Community-wide setting in Kampala Uganda / Female Adult	Presents the study protocol for a pair-matched cluster randomized trial designed to assess the community-level impact of the SASA! Intervention to prevent violence against women and reduce HIV/AIDS risk in eight communities of Kampala, Uganda. It is one of the few cluster randomized trials to assess the impact of a gender-focused community mobilization intervention.
Scott 2013a^39^ / Original research, mixed	Non-displaced populations / Community setting in Eastern Democratic Republic of Congo / General	Describes a mixed methods assessment to assess community attitudes and health facility capacity to address GBV in eastern Democratic Republic of Congo. While the majority of respondents favored legal action over community mediation to obtain justice for GBV, more than half of survivors interviewed reported being forced to accept community mediation.
Slegh 2013^40^ / Original research, qualitative	Non-displaced populations / Rwanda / Female Adult	Presents an evaluation of a pilot project in Rwanda that deliberately engaged men as partners of women beneficiaries of a micro-credit program. Preliminary results affirm the importance of engaging men in a deliberate questioning of gender norms and power dynamics so that they can embrace better co-operation and sharing of activities at the household level; and that a ‘do-no-harm’ approach to women’s economic empowerment should involve activities to engage men at the community level in questioning and ending GBV – building on those interventions that have shown evidence of changes in men’s attitudes and behaviors related to GBV.
Lwambo 2013^41^ / Original research, qualitative	Non-displaced populations / North Kivu province in Democratic Republic of Congo / Female Adult	Draws on a research study on the discrepancies between dominant ideals of masculinities and the actual realities of men’s lives to reflect on the relationship between GBV and conceptions of masculinity in conflict affected North Kivu province of the Democratic Republic of Congo. The author argues that GBV interventions that focus exclusively on women do not recognize the interdependent and interactive nature of gender and emphasizes the need for holistic approaches that empower men to make non-violent life choices.
Hoang 2013^42^ / Original research, qualitative	Non-displaced populations / Coastal district in Vietnam / Female Adult	Describes a pilot GBV prevention project that worked with men in a coastal district in Vietnam to stop violence against their wives, enabling them to develop positive ideas about what it is to be a man and empowering them to adopt these new values in their thoughts and practices.
Mitchell 2013^43^ / Commentary	Non-Displaced Population / Community education case study in peri-urban area of Lima, Peru / Female Adult	A case study of a project using a community education approach to challenge stereotypes about gender roles, question men’s assumed dominance over women and support men to construct new forms of masculinity without violence in urban Peru.
Scott 2013b^44^ / Original research, quantitative	Non-displaced populations / Selected cities (n=7) located in South Sudan / Female Adult	Describes a community-based participatory research study of attitudes towards gender inequitable norms related to GBV in South Sudan. It found that both men and women agreed with gender-inequitable norms, further supporting that GBV programming should address the attitudes of both women and men and supporting education promotion as a strategy for addressing GBV.
Asgary 2013^45^ / Review	Displaced populations / Evaluation studies in a variety of geographical areas / General	Reviews peer-reviewed studies on evaluation of strategies for prevention and management of GBV among refugee populations before September 2011. Studies not primarily focused on prevention and treatment, and not describing a population, health outcome, and interventions, were excluded.
Spangaro 2013^46^ / Review	Displaced populations / Impact evaluations in a variety of geographical areas / Female Adult	Reviews the extent and impact of initiatives to reduce incidence, risk and harm from sexual violence in conflict, post-conflict and other humanitarian crisis, in low and middle-income countries. The reviewed examined studies across seven domains: survivor care, livelihood initiatives, community mobilization, personnel initiatives, systems and security responses, legal interventions, and multiple component interventions.
Abdelnour 2014^47^ / Original research, qualitative	Displaced populations / Camp settings in Darfur, Sudan / Female Adult	Examines the use of fuel-efficient stoves in humanitarian space and considers its effect on reducing sexual violence as well as other effects not yet considered. The concept is based on the premise that firewood collection by women and girls increases risk of sexual violence.
Hossain 2014^48^ / Original research, quantitative	Non-displaced populations / 12 pair-matched communities in Cote d’Ivoire / Female Adult	Evaluates the impact of adding a targeted men’s intervention to a community-based prevention program in Cote d’Ivoire aimed at reducing intimate partner violence.
Gurman 2014^49^ / Original research, qualitative	Displaced populations / Post-conflict settings in South Sudan, Uganda, Thailand, Liberia and Rwanda / Female Adult	Examines the, “Through Our Eyes”, intervention, which is a multi-year participatory video project aimed at reducing GBV in post-conflict settings in South Sudan, Uganda, Thailand, Liberia, and Rwanda. The study utilizes a qualitative analysis design for evaluating the effectiveness of the project.
Spangaro 2015^50^ / Review	Displaced populations / Outcome evaluations in a variety of geographical areas / Female Adult & Female Child	This article uses an exploratory theory-driven method for conducting a systematic review examining the mechanisms that contribute to GBV related outcomes. Four main mechanisms appear to contribute to effective interventions: increasing the risk to offenders of being detected; building community engagement; ensuring community members are aware of available help for and responses to sexual violence; and safe and anonymous systems for reporting and seeking help.

Key findings pertaining to the peer-reviewed literature (i.e. studies published in peer-reviewed journals included in this review), organized by types of prevention programming, are as follows:

**(1) Transforming socio-cultural norms, with an emphasis on empowering women and girls**

None of the peer-reviewed articles identified through the systematic search addressed gender transformative programming or presented research on interventions seeking to address socio-cultural norms in refugee settings. A number of studies in other settings, however, sought to gain an understanding of gender norms and evaluate gender-transformative interventions, specifically addressing conceptions of masculinity seen as factors in GBV. For example, one community-based study in South Sudan found that both men and women agreed with gender-inequitable norms, for example, husband has the right to demand sex from wife, men need more sex then women, it is more important to educate boys than girls, which provides further support for GBV programming addressing the attitudes of both women and men 44. Six additional articles evaluating or reflecting on interventions to transform socio-cultural norms are summarized below in the sub-section on engaging men and boys in GBV prevention and response. Finally, one article evaluated a multi-year participatory video project aimed at reducing GBV in post-conflict settings and found that the intervention was associated with increased awareness of women’s rights and gender equality, as well as a reduction in the culture of silence on GBV, and enhanced empowerment of survivors to seek health care and legal services 49. However, the study did not provide any indication as to the effectiveness of the intervention; more rigorous evaluation of this intervention would be required to understand its potential impact.

**(2) Rebuilding family and community structures and support systems**

None of the peer-reviewed articles specifically addressed an intervention aimed at family and community structures and support systems in refugee or non-refugee settings. However, two studies 46 ^,^ 50 did cite the importance of building local capacity and community engagement in efforts to reduce the incidence of GBV.

**(3) Creating conditions to improve accountability systems**

Three of the ten peer-reviewed articles 33 ^,^ 36 ^,^ 45 addressed accountability issues in displaced populations, but none presented proven strategies for effective GBV prevention. One study explored refugees’ perspectives on GBV and systems for holding perpetrators accountable in a Rwanda refugee camp. The study found that even when incident monitoring and response services are available, social norms and power dynamics within refugee communities may prevent women from accessing them. The study concluded that a top-down approach to GBV prevention may reinforce disparities by affording the same actors who exploit community members the power to define and control monitoring, response, and evaluation processes. To effectively improve accountability systems, study participants suggested collaborative programs that engage camp leaders, non-governmental organization (NGO) staff, and residents in shared problem solving around culturally appropriate responses to human rights abuses 36. Similarly, a qualitative study of formal and informal structures to address intimate partner violence (IPV) in the Kakuma refugee camp in Kenya found that UNHCR community-based efforts had not fully engaged with community members in developing a response to IPV. Refugees spoke about making use of a “hierarchy of responses,” only utilizing resources put in place by international agencies when a situation is found to be beyond the capacity of a community. The authors presented a number of reasons why women may prefer community response mechanisms to those of international agencies, including male distrust of agency responses and female fear of estrangement from community support networks. While community responses might often be appropriate and helpful, the study suggests that in Kakuma they do not necessarily result in women receiving protection and preventing further violence. In some cases, women were advised to return to a violent partner and modify their behavior in order to avoid provoking further violence 33. Lastly, a systematic review of prevention and management strategies for the consequences of GBV in refugee settings identified research into the efficacy and effectiveness of various GBV methods as an obstacle to achieving effective accountability systems in camp settings 45.

**(4) Designing effective services and facilities**

None of the articles reviewed present proven or well-established practices for effectively preventing GBV in refugee settings, and one of the studies indicated that the single biggest barrier to implementation was the absence of evidence-based interventions 45. One study 46 found that while a majority of GBV interventions addressing sexual violence in humanitarian crises were implemented in conflict and post-conflict settings, most literature focused on sexual violence by combatants and provided little information on sexual violence by partners. The review also noted that no prospective studies on sexual violence incidence were associated with interventions had been published, though several studies did indicate a reduction in women’s exposure to sexual violence associated with firewood distribution to reduce the need for women and girls having to leave camps to search for firewood. Related to risks associated with firewood collection, on the issue of distribution of fuel-efficient stoves, one study 47, found a lack of evidence to support the intervention and suggested that such an intervention could inadvertently place a larger economic burden on women as they needed to have access to fuel for cooking.

Two of the ten peer-reviewed articles in displacement settings discuss gaps in reproductive health service provision, including gaps in adherence to international guidelines and standards for prevention and response to GBV 29 ^,^ 30. Although neither article directly focuses on primary prevention programming, both identify barriers to utilization of clinical response services for survivors of sexual violence that could be informative for the design of effective multi-sectoral services and facilities for displaced populations. For example, both studies of Iraqi refugees in Jordan 29 and GBV survivors in Northern Uganda 30 found limited awareness about the availability of response services, suggesting educational campaigns may help inform the refugee community about the urgency of clinical care to prevent negative health outcomes. A review of sexual violence in humanitarian emergencies 28 emphasized the urgency of establishing coordinated multi-sectoral GBV prevention and response services in emergencies even if there is not substantial data on the prevalence of the problem in the targeted setting. Multiple component interventions and community engagement were also cited as contributing to positive outcomes for GBV survivors 46.

There is a limited but growing body of evidence on effective GBV prevention models in non-refugee settings. The most commonly referenced example is the Intervention with Microfinance for AIDS & Gender Equity (IMAGE), which combines microfinance with a participatory gender and HIV training curriculum for rural women in Limpopo Province, South Africa. An evaluation of the intervention found that program participants experienced a 55% reduction in physical and/or sexual violence by an intimate partner relative 31. Related studies, also conducted within South Africa, found the intervention to be cost effective, feasible to deliver, and acceptable to most participants 34 ^,^ 35. These evaluations suggest that even in the short term, reductions in GBV might be achievable through structural development initiatives such as microfinance if combined with culturally appropriate initiatives to address the changes in socio-economic dynamics that may result from women’s empowerment through the program.

As indicated in one study 46 increased risk of GBV is associated with lack of protection, stigma, and retaliation associated with interventions; therefore, evidence points to the need for interventions that build on local capacity, while avoiding risk and re-traumatization to survivors. A systematic review of literature on mechanisms contributing to effective interventions 50 found that four mechanisms were particularly important: (1) increasing risk to offenders of being detected; (2) building community engagement; (3) ensuring community members are aware of available help for and responses to sexual violence; and (4) safe and anonymous systems for reporting and seeking help. These mechanisms contribute most to multiple component interventions as well as those pertaining to firewood collection and codes of conduct for personnel and legal issues. However, increasing risk of detection of offenders only appears to be effective in the context of firewood collection, while the other three mechanisms appear to be more general and seem to work best when carried out simultaneously.

**(5) Working with formal and traditional legal systems**

None of the peer-reviewed articles focus on working with formal and traditional legal systems in refugee settings. However, one article 49 evaluated a participatory video project that sought to encourage GBV survivors to seek legal services among displaced populations in post-conflict settings. Another article 39 discusses community attitudes toward formal legal action to address GBV in non-displaced populations in conflict-affected eastern Democratic Republic of Congo (DRC) and provides additional examples to highlight the complexity of addressing accountability. Their mixed methods study found respondents agreed that perpetrators should be punished and survivors compensated, and a majority of respondents favoured use of the legal system to obtain justice. Quantitative and qualitative findings both suggest that GBV survivors might be forced to use community-mediated systems, calling into question whether appropriate punishment or compensation could be obtained.

**(6) Assessment, monitoring, and documentation of GBV**

None of the articles reviewed present methods for measuring or evaluating the effectiveness of GBV prevention programming in refugee settings, though several articles did address barriers as well as enabling factors for documentation of GBV. The two articles assessing health service provision for GBV survivors 29 ^,^ 30 demonstrate how international guidelines and standards, specifically the 2005 IASC Guidelines for GBV Interventions in Humanitarian Settings can be used as the foundation for assessment tools. New guidelines for GBV interventions in humanitarian settings were published in late 2015; have yet to be evaluated in the scientific literature, and are therefore, outside the purview of this review.^51^ A third article that reviewed sexual violence in humanitarian emergencies 28 identified barriers to conducting population-based research on GBV, including the political implications of drawing attention to the occurrence of sexual violence in conflict settings and potential risks to both researchers and respondents participating in research efforts. A fourth article 50 stressed the need for anonymous systems for reporting and seeking help, otherwise many GBV events will remain undocumented.

**(7) Engaging men and boys in GBV prevention and response**

None of the peer-reviewed articles focus on engaging refugee men and boys in GBV prevention and response. However, there is a limited but growing body of evidence on engaging men and boys in GBV prevention and response in other settings. Impact and process evaluations of programs using participatory group education to change norms associated with violence have shown positive results; some of these programs involve women and men to change norms associated with GBV 32. The general conclusion from impact evaluations of multi-faceted programs with microfinance components is that adding such evidence-based GBV prevention activities —both in the form of group education and community-based campaign activism— can be relatively easily combined with women’s economic empowerment activities when adequate resources and training available 31 ^,^ 34 ^,^ 35 ^,^ 40.

The available research also suggests that programs aiming to empower women economically should be attentive to the possibility that men who perceive themselves to be economically vulnerable or marginalized are already, in some settings, more likely to commit GBV. If economically marginalized men view their traditional roles as “heads of households” being eroded by women’s income-generating activities, engaging men in a deliberate questioning of such roles can enable them to embrace cooperation and sharing of activities at the household level 40. In addition, women-focused economic empowerment programs could include community campaigns targeting men and training for government and NGO staff on ways to engage men as partners.

A 2006 a review of 58 evaluations of engaging men and boys in GBV prevention and health programming found that programs that promote gender-equitable relationships between men and women by engaging men in discussions of gender and masculinity with deliberate efforts to transform gender norms may be more effective in producing behavior change than more narrowly focused interventions that merely acknowledge gender norms and roles 32. These guiding principles, and the complexities of putting these strategies into practice, are further supported by evidence presented in a 2013 series of articles documenting programs that engage men as allies in GBV prevention by facilitating a deliberate questioning of gender norms and power dynamics in the DRC, Rwanda, Peru, and Vietnam 40 ^,^ 41 ^,^ 42 ^,^ 43. First, the evaluation of a pilot project deliberately engaging men as partners of female beneficiaries of CARE Rwanda’s Village Savings and Loan program affirmed the importance of men’s involvement in household cooperation and sharing of activities 40. Another article draws on a research study of the discrepancies between dominant ideals of masculinity and the actual realities of men’s lives in the eastern DRC. Respondents were critical of the fact that most GBV prevention and response programs focus exclusively on women and drew a direct connection between the resulting sense of failure and unhealthy outlets for asserting masculinity, lack of productivity, and GBV 41. The article described a pilot project working with men to prevent violence against women in a coastal district in Vietnam. The findings illustrate the challenges of reducing society’s tolerance of violence against women without increasing stigmatization of and objections to men in general and male perpetrators of GBV in particular. The authors found that men need knowledge, skills, mentoring, and peer support to construct a positive, non-violent version of masculinity 42. The case study from Peru presents a model for challenging men’s deeply held gender norms that are considered to be a causal factor for domestic violence. Elements presented in the case study that contribute to the program’s success include a “horizontal” relationship between facilitators and participants, mutual learning approaches to group education with optional individual counseling sessions, and engagement of the family through community worker visits to create a more integrated intervention 43. An additional study, evaluating the effectiveness of adding a 16-session Men’s Discussion Group intervention to a community-based prevention program in Cote d’Ivoire, specifically a 16-session Men’s Discussion Group intervention, found that the targeted intervention significantly influenced men’s reported behaviors related to hostility and conflict management, and suggested that concerted efforts to include men in GBV prevention programming could reduce intimate partner violence in conflict-affected settings 48.

## Discussion

There is a limited body of evidence available on effective GBV prevention strategies, especially in refugee settings. This gap is more pronounced with regards to prevention of forms of GBV other than sexual violence as an act of war, including intimate partner violence. Few of the articles reviewed provided objective data to showing the effectiveness of interventions and findings of those that did may not be generalizable. None of the peer-reviewed articles identified in the review presented proven strategies for addressing socio-cultural norms, rebuilding family and community support structures, improving accountability systems, designing effective services and facilities, working with formal and traditional legal systems, monitoring and documenting GBV, or engaging men and boys in GBV prevention and response in refugee settings.

This evidence gap may be related to both the challenge of conducting research on GBV prevention efforts and difficulties of conducting impact evaluations in humanitarian settings. For example, there are ethical considerations in conducting long-term evaluation of displaced populations, and shifting organizational priorities and donor funding may limit the duration of GBV programs. Additionally, security and logistical challenges are typically present in refugee settings. Limitations in the availability of staff, training, expertise, and funding for GBV programs in displacement settings are also common 52 ^,^ 53. Further research and consideration would be needed to determine the generalizability of findings on effective male engagement, group education, and economic empowerment strategies to prevent GBV in refugee populations. Rigorous evaluations of GBV prevention efforts in refugee populations, where the risk of GBV may be highest, are critically needed.

## Limitations

As indicated by previous GBV reviews, and particularly those focused on sexual violence against women and girls, there remains a clear gap in the quality and availability of impact evaluations for past and present interventions focused on GBV prevention more broadly, and this gap is even more pronounced for refugee populations. As a consequence of the general lack of published literature on GBV prevention in refugee populations, the inclusion of articles focused on GBV prevention in non-refugee populations (including both internally displaced and non-displaced populations) was considered necessary. Evaluations of GBV prevention efforts in non-refugee settings may not be generalizable to refugee populations where the risk profile and contextual factors may be different. Given the varied populations, interventions, and study designs of included studies, findings were given equal consideration in analysis, regardless of the study design, quality or potential risk of bias.

## Conclusion

This study provides an integrative review of scientific publications related to GBV prevention in refugee populations that goes beyond the scope of previous reviews and compares the availability of evidence for different types of GBV prevention activities recommended by the global humanitarian community. Whereas previous reviews addressing refugee populations has focused on conflict related sexual violence, this study expands scope of the literature to review socio-cultural norms change, rebuilding family and community support structures, improving accountability systems, designing effective services and facilities, working with formal and traditional legal systems, monitoring and documenting GBV, and/or engaging men and boys in GBV prevention and response in refugee settings. Upon examination, there were few studies providing evidence of effective GBV prevention strategies or interventions, suggesting that there is little research conducted on the effectiveness of GBV prevention efforts in humanitarian settings or that evaluations of these programs are rarely published in peer-reviewed scientific literature. This supports Asgary et al’s findings 45 that guidelines for GBV prevention in humanitarian settings are either based on expert opinion or extrapolation of evidence from non-emergency affected populations. In either case, the lack of evidence in the peer-reviewed literature limits the ability to learn from the experience of previous programs or interventions. Commonly agreed upon standards or guidelines for evaluation of GBV prevention programming, and publication of evaluations conducted using these guidelines, could assist humanitarian stakeholders to build and disseminate an evidence base of effective GBV prevention interventions, programs and strategies. Evaluation of GBV prevention efforts, especially among refugee populations, must be given higher priority to justify continuation or revision of recommended GBV activities/programs being implemented in diverse humanitarian settings. Evaluation of known and applied GBV prevention strategies identified in this review should be a priority of the global humanitarian community.

## Competing Interests

The authors have declared that no competing interests exist.

## References

[1] Vu, A., Adam, A., Wirtz, A., Pham, K., Rubenstein, L., Glass, N., ... & Singh, S. (2014). The prevalence of sexual violence among female refugees in complex humanitarian emergencies: a systematic review and meta-analysis. PLoS currents, 6. 10.1371/currents.dis.835f10778fd80ae031aac12d3b533ca7 PMC4012695 24818066

[2] United Nations. Division for the Advancement of Women. (2000). Convention on the Elimination of All Forms of Discrimination against Women, the Optional Protocol: text and materials. United Nations Publications.

[3] Sullivan, D. J. (1994). Women's Human Rights and the 1993 World Conference on Human Rights. American Journal of International Law, 152-167.

[4] World Health Organization. (2013). Global and regional estimates of violence against women: prevalence and health effects of intimate partner violence and non-partner sexual violence. World Health Organization.

[5] UNHCR. (1999). Reproductive Health in Refugee Situations: an Inter-agency Field Manual. Geneva, Switzerland. United Nations High Commissioner on Refugees.

[6] Austin, J., Guy, S., Lee-Jones, L., McGinn, T., & Schlecht, J. (2008). Reproductive health: a right for refugees and internally displaced persons. Reproductive Health Matters, 16(31), 10-21. 10.1016/S0968-8080(08)31351-2 18513603

[7] Shanks, L., & Schull, M. J. (2000). Rape in war: the humanitarian response. Canadian Medical Association Journal, 163(9), 1152-1156. PMC80250 11079062

[8] Buscher, D. (2006). Displaced women and girls at risk: risk factors protection solutions and resource tools.

[9] UNHCR. (2014). Global Trends: Forced Displacement in 2014. Geneva, Switzerland. United Nations High Commissioner on Refugees.

[10] UNHCR. (2013). Global Trends: Forced Displacement in 2013. Geneva, Switzerland. United Nations High Commissioner on Refugees.

[11] Syria Regional Refugee Response. (2015, December 31). Retrieved from http://data.unhcr.org/syrianrefugees/regional.php#_ga=1.131453964.2090026747.1446427142

[12] Parker, S. (2015). Hidden crisis: violence against Syrian female refugees. The Lancet, 385(9985), 2341-2342. 10.1016/S0140-6736(15)61091-1 26088630

[13] United Nations Security Council. (2009). United Nations Security Council Resolution 1889. New York, NY. United Nations.

[14] United Nations Secretary General. (2006). Statement of commitment on eliminating sexual exploitation and abuse by UN and non-UN personnel. New York, NY. United Nations.

[15] Kim, A. A., Malele, F., Kaiser, R., Mama, N., Kinkela, T., Mantshumba, J. C., ... & Reed, K. H. (2009). HIV infection among internally displaced women and women residing in river populations along the Congo River, Democratic Republic of Congo. AIDS and Behavior, 13(5), 914-920. 10.1007/s10461-009-9536-z 19319674

[16] Spiegel, P. B., Bennedsen, A. R., Claass, J., Bruns, L., Patterson, N., Yiweza, D., & Schilperoord, M. (2007). Prevalence of HIV infection in conflict-affected and displaced people in seven sub-Saharan African countries: a systematic review. The Lancet, 369(9580), 2187-2195. 10.1016/S0140-6736(07)61015-0 17604801

[17] Lehmann, A. (2002). Safe abortion: a right for refugees?. Reproductive Health Matters, 10(19), 151-155. 10.1016/s0968-8080(02)00026-5 12369319

[18] John-Langba, J. (2007). The relationship of sexual and gender-based violence to sexual-risk behaviour among refugee women in Sub-Saharan Africa. World health & population, 9(2), 26-37. 10.12927/whp.2007.18957 18270504

[19] Sideris, T. (2003). War, gender and culture: Mozambican women refugees. Social science & medicine, 56(4), 713-724. 10.1016/s0277-9536(02)00067-9 12560006

[20] UNICEF. (2003). Sexual and Gender-Based Violence against Refugees, Returnees and Internally displaced Persons-Guidelines for Prevention and Response.

[21] Barker, G. T., Ricardo, C., & Nascimento, M. (2007). Engaging Men and Boys in Changing Gender-based Inequity in Health: Evidence from Programme Intevrentions. Geneva: World Health Organization.

[22] Harvey, A., Garcia-Moreno, C., & Butchart, A. (2007). Primary prevention of intimate partner violence and sexual violence: Background paper for WHO expert meeting May 2-3, 2007. Geneva: World Health Organization, Department of Violence and Injuy Prevention and Disability.

[23] World Health Organization (WHO). (2012). Executive summary: a research agenda for sexual violence in humanitarian, conflict and post-conflict settings. World Health Organization.

[24] Hossain, M., Zimmerman, C., & Watts, C. (2014). Preventing violence against women and girls in conflict. The Lancet, 383(9934), 2021-2022. 10.1016/S0140-6736(14)60964-8 24923526

[25] Arango, D. J., Morton, M., Gennari, F., Kiplesund, S., & Ellsberg, M. (2014). Interventions to Prevent or Reduce Violence Against Women and Girls: A Systematic Review of Reviews.

[26] Spangaro, J., Adogu, C., Zwi, A. B., Ranmuthugala, G., & Davies, G. P. (2015). Mechanisms underpinning interventions to reduce sexual violence in armed conflict: A realist-informed systematic review. Conflict and health, 9(1), 1-14. 10.1186/s13031-015-0047-4 PMC4499895 26170898

[27] Evaluation of Implementation of 2005 IASC Guidelines for Gender-based Violence Interventions in Humanitarian Settings in the Syria Crisis Response. (2015, October 11). Retrieved from http://reliefweb.int/report/syrian-arab-republic/evaluation-implementation-2005-iasc-guidelines-gender-based-violence

[28] Marsh, M., Purdin, S., & Navani, S. (2006). Addressing sexual violence in humanitarian emergencies. Global Public Health, 1(2), 133-146. 10.1080/17441690600652787 19153902

[29] Chynoweth, S. K. (2008). The need for priority reproductive health services for displaced Iraqi women and girls. Reproductive Health Matters, 16(31), 93-102. 10.1016/S0968-8080(08)31348-2 18513611

[30] Henttonen, M., Watts, C., Roberts, B., Kaducu, F., & Borchert, M. (2008). Health services for survivors of gender-based violence in northern Uganda: a qualitative study. Reproductive Health Matters, 16(31), 122-131. 10.1016/S0968-8080(08)31353-6 18513614

[31] Pronyk, P. M., Hargreaves, J. R., Kim, J. C., Morison, L. A., Phetla, G., Watts, C., ... & Porter, J. D. (2006). Effect of a structural intervention for the prevention of intimate-partner violence and HIV in rural South Africa: a cluster randomised trial. The lancet, 368(9551), 1973-1983. 10.1016/S0140-6736(06)69744-4 17141704

[32] Barker, G., Ricardo, C., Nascimento, M., Olukoya, A., & Santos, C. (2010). Questioning gender norms with men to improve health outcomes: evidence of impact. Global Public Health, 5(5), 539-553. 10.1080/17441690902942464 19513910

[33] Horn, R. (2010). Responses to intimate partner violence in Kakuma refugee camp: refugee interactions with agency systems. Social science & medicine, 70(1), 160-168. 10.1016/j.socscimed.2009.09.036 19846247

[34] Hargreaves, J., Hatcher, A., Strange, V., Phetla, G., Busza, J., Kim, J., ... & Bonell, C. (2009). Process evaluation of the Intervention with Microfinance for AIDS and Gender Equity (IMAGE) in rural South Africa. Health Education Research, cyp054. 10.1093/her/cyp054 19797337

[35] Jan, S., Ferrari, G., Watts, C. H., Hargreaves, J. R., Kim, J. C., Phetla, G., ... & Pronyk, P. M. (2011). Economic evaluation of a combined microfinance and gender training intervention for the prevention of intimate partner violence in rural South Africa. Health policy and planning, 26(5), 366-372. 10.1093/heapol/czq071 20974751

[36] Ho, A., & Pavlish, C. (2011). Indivisibility of accountability and empowerment in tackling gender-based violence: Lessons from a refugee camp in Rwanda. Journal of Refugee Studies, 24(1), 88-109.

[37] Sikweyiya, Y., & Jewkes, R. (2011). Perceptions about safety and risks in gender-based violence research: implications for the ethics review process. Culture, health & sexuality, 13(9), 1091-1102. 10.1080/13691058.2011.604429 21824018

[38] Abramsky, T., Devries, K., Kiss, L., Francisco, L., Nakuti, J., Musuya, T., ... & Watts, C. (2012). A community mobilisation intervention to prevent violence against women and reduce HIV/AIDS risk in Kampala, Uganda (the SASA! Study): study protocol for a cluster randomised controlled trial. Trials, 13(1), 96. 10.1186/1745-6215-13-96 PMC3503643 22747846

[39] Scott, J., Polak, S., Kisielewski, M., McGraw‐Gross, M., Johnson, K., Hendrickson, M., & Lawry, L. (2013a). A mixed‐methods assessment of sexual and gender‐based violence in eastern Democratic Republic of Congo to inform national and international strategy implementation. The International journal of health planning and management, 28(3), e188-e216. 10.1002/hpm.2144 23108942

[40] Slegh, H., Barker, G., Kimonyo, A., Ndolimana, P., & Bannerman, M. (2013). ‘I can do women's work’: reflections on engaging men as allies in women's economic empowerment in Rwanda. Gender & Development, 21(1), 15-30.

[41] Lwambo, D. (2013). ‘Before the war, I was a man’: men and masculinities in the Eastern Democratic Republic of Congo. Gender & Development, 21(1), 47-66.

[42] Hoang, T. A., Quach, T. T., & Tran, T. T. (2013). ‘Because I am a man, I should be gentle to my wife and my children’: positive masculinity to stop gender-based violence in a coastal district in Vietnam. Gender & Development, 21(1), 81-96.

[43] Mitchell, R. (2013). Domestic violence prevention through the Constructing Violence-free Masculinities programme: an experience from Peru. Gender & Development, 21(1), 97-109.

[44] Scott, J., Averbach, S., Modest, A. M., Hacker, M. R., Cornish, S., Spencer, D., ... & Parmar, P. (2013b). An assessment of gender inequitable norms and gender-based violence in South Sudan: a community-based participatory research approach. Confl Health, 7(4). 10.1186/1752-1505-7-4 PMC3599371 23497469

[45] Asgary, R., Emery, E., & Wong, M. (2013). Systematic review of prevention and management strategies for the consequences of gender-based violence in refugee settings. International health, 5(2), 85-91. 10.1093/inthealth/iht009 24030107

[46] Spangaro, J., Adogu, C., Ranmuthugala, G., Davies, G. P., Steinacker, L., & Zwi, A. (2013). What evidence exists for initiatives to reduce risk and incidence of sexual violence in armed conflict and other humanitarian crises? A systematic review. 10.1371/journal.pone.0062600 PMC3655168 23690945

[47] Abdelnour, S., & Saeed, A. M. (2014). Technologizing Humanitarian Space: Darfur Advocacy and the Rape‐Stove Panacea. International Political Sociology, 8(2), 145-163.

[48] Hossain, M., Zimmerman, C., Kiss, L., Abramsky, T., Kone, D., Bakayoko-Topolska, M., ... & Watts, C. (2014). Working with men to prevent intimate partner violence in a conflict-affected setting: a pilot cluster randomized controlled trial in rural Côte d’Ivoire. BMC public health, 14(1), 339. 10.1186/1471-2458-14-339 PMC4007144 24716478

[49] Gurman, T. A., Trappler, R. M., Acosta, A., McCray, P. A., Cooper, C. M., & Goodsmith, L. (2014). ‘By seeing with our own eyes, it can remain in our mind’: qualitative evaluation findings suggest the ability of participatory video to reduce gender-based violence in conflict-affected settings. Health education research, cyu018. 10.1093/her/cyu018 24973224

[50] Spangaro, J., Adogu, C., Zwi, A. B., Ranmuthugala, G., & Davies, G. P. (2015). Mechanisms underpinning interventions to reduce sexual violence in armed conflict: A realist-informed systematic review. Conflict and health, 9(1), 1-14. 10.1186/s13031-015-0047-4 PMC4499895 26170898

[51] Inter-Agency Standing Committee. (2005). Guidelines for gender-based violence interventions in humanitarian settings: Focusing on prevention of and response to sexual violence in emergencies. In Guidelines for gender-based violence interventions in humanitarian settings: Focusing on prevention of and response to sexual violence in emergencies.

[52] Clarke, M., Allen, C., Archer, F., Wong, D., Eriksson, A., & Puri, J. (2014). What evidence is available and what is required, in humanitarian assistance. International Initiative for Impact Evaluation, London.

[53] Puri, J., Aladysheva, A., Iversen, V., Ghorpade, Y., & Bruck, T. (2015). What methods may be used in impact evaluations of humanitarian assistance. Institute for the Study of Labor, Bonn.

